# Analyzing the impact of unemployment on mental health among Chinese university graduates: a study of emotional and linguistic patterns on Weibo

**DOI:** 10.3389/fpubh.2024.1337859

**Published:** 2024-05-09

**Authors:** Miaoqing Tan, Zhigang Wu, Jin Li, Yuxi Liang, Wenting Lv

**Affiliations:** Guangzhou Sport University, Guangzhou, China

**Keywords:** unemployment rates, anxiety expressions, linguistic features, university students, Sina Weibo

## Abstract

**Purpose:**

This study explores the intricate relationship between unemployment rates and emotional responses among Chinese university graduates, analyzing how these factors correlate with specific linguistic features on the popular social media platform Sina Weibo. The goal is to uncover patterns that elucidate the psychological and emotional dimensions of unemployment challenges among this demographic.

**Methods:**

The analysis utilized a dataset of 30,540 Sina Weibo posts containing specific keywords related to unemployment and anxiety, collected from January 2019 to June 2023. The posts were pre-processed to eliminate noise and refine the data quality. Linear regression and textual analyses were employed to identify correlations between unemployment rates for individuals aged 16–24 and the linguistic characteristics of the posts.

**Results:**

The study found significant fluctuations in urban youth unemployment rates, peaking at 21.3% in June 2023. A corresponding increase in anxiety-related expressions was noted in the social media posts, with peak expressions aligning with high unemployment rates. Linguistic analysis revealed that the category of “Affect” showed a strong positive correlation with unemployment rates, indicating increased emotional expression alongside rising unemployment. Other categories such as “Negative emotion” and “Sadness” also showed significant correlations, highlighting a robust relationship between economic challenges and emotional distress.

**Conclusion:**

The findings underscore the profound impact of unemployment on the emotional well-being of university students, suggesting that economic hardships are closely linked to psychological stress and heightened negative emotions. This study contributes to a holistic understanding of the socio-economic challenges faced by young adults, advocating for comprehensive support systems that address both the economic and psychological facets of unemployment.

## 1 Introduction

Over the recent past, the escalating rate of unemployment among Chinese youth has been a matter of concern, drawing attention to an evolving crisis with profound socio-economic implications. Urbanization, evolving economic structures, and swift technological advancements have significantly reshaped the labor market dynamics, exacerbating the challenge of integrating fresh graduates into the workforce. This conundrum is most evident in the burgeoning number of university graduates grappling with underemployment or outright unemployment - a situation that has swiftly evolved into a national concern. The depth of this quandary is underscored by the stark unemployment statistics among Chinese youth aged 16–24. Over recent months, the unemployment rate within this demographic has consistently breached the 20% mark, a disconcerting trend that attests to the urgency of the issue. Specifically, in June 2023, the youth unemployment rate soared to 21.3%, setting a record high for the third consecutive month. Notably, the months of June and July coincide with the peak of the graduation season, where a notable influx of young individuals enters the job market. Over the past few years, the momentum behind this trend has been particularly swift due to the rapidly increasing number of university graduates (1, 2).

Unemployment is far more than a simple economic issue; it is deeply entwined with public health concerns. The adverse effects on psychological well-being, particularly the anxiety associated with joblessness, have been well-documented in numerous studies over recent decades. For instance, Paul and Moser (2009) conducted an extensive longitudinal study that demonstrated a strong link between sharp increases in unemployment rates and heightened anxiety levels among impacted populations (3). Similarly, a cross-sectional survey conducted by BaticMujanovic et al., indicated that prolonged unemployment not only fosters heightened anxiety but also predisposes individuals to chronic stress-related disorders (4). Moreover, their findings indicated a direct correlation between the duration of unemployment and increased severity of anxiety symptoms. Furthermore, socio-economic factors play an undeniable role in this sphere. Smith et al. elucidated that marginalized communities and those with lower educational qualifications often bear a disproportionately higher burden of anxiety due to unemployment, further emphasizing the need for targeted interventions in these communities (5). Additionally, the broader societal implications of unemployment-induced anxiety were explored by Wangmo (6). Their multi-disciplinary approach delineated how increased joblessness rates not only impact individual mental well-being but also manifest in community-level social unrest, further exacerbating the socio-economic challenges faced by these communities.

The complex relationship between unemployment and mental health is emerging as an increasingly significant area of study, with a growing body of literature underscoring a direct correlation between these two factors (7). Unemployment is far more than an economic condition; it is a state of being that, left unresolved, can give rise to psychological distress, dwindling self-esteem, and escalating anxiety levels (3). This nexus is particularly poignant for university students, who, standing at the threshold of their professional lives, are saddled with aspirations for the future and societal and familial expectations. Their transition from academia to the professional world is often marked by uncertainty and stress, which are significantly amplified in the face of unemployment or underemployment (8). Given the profound implications of Unemployed Chinese university students, it is no longer enough to approach this issue from an economic perspective alone. Instead, a multidisciplinary lens is required to comprehend the full scope of its impact, particularly the psychological toll it exacts on the individuals affected. Unemployment in early career stages can have enduring effects on mental health, leading to increased vulnerability to various psychological disorders, including anxiety and depression (9).

Public perception towards anxiety disorders can significantly impact the psychological well-being and everyday functioning of affected individuals. To date, a considerable body of research has been devoted to understanding attitudes towards mental illnesses, predominantly employing questionnaire-based surveys as the primary method of data collection (10–12). However, current research methodologies, including questionnaires, may be subject to investigator bias, with their design and interpretation heavily influenced by researchers’ subjectivity. Moreover, these traditional methods lack the capability to delve into the nuanced structure of language expression, an aspect central to reflecting an individual’s cognitive processes and inner activities (13). As we strive to uncover insights about the language used by broad demographics, we find ourselves increasingly drawn to the wealth of information that social media platforms provide. Serving as key channels for mass communication, these platforms host an abundance of information, offering a reflection of people’s inner workings and emotional states. It is not uncommon for users to anonymously disclose their mental challenges or diseases across a diverse range of social media and online health communities (14). Such virtual health ecosystems can serve as a refuge of empathy, promoting interactions among those experiencing similar symptoms (15). Furthermore, users frequently leverage these platforms to gather health information pertaining to their symptoms in their journey towards self-diagnosis (16). Therefore, the discourse unfolding on social media serves as a natural linguistic source, mirroring societal perspectives on anxiety.

Weibo’s widespread usage among Chinese university students makes it an effective and practical tool for investigating the underexplored dimensions of their employment-related anxieties (17). The platform’s anonymity feature allows users to express their thoughts candidly, reducing the likelihood of self-censorship that may arise in face-to-face interviews or surveys (18). Consequently, Weibo provides a naturalistic, non-intrusive means to access the nuanced anxieties and expectations of Chinese graduates concerning employment. Moreover, the use of Weibo as a data source circumvents some of the limitations associated with traditional data collection methods, such as nonresponse and recall bias, enhancing the reliability and validity of the insights generated (19). The vast amount of data generated on the platform also allows for longitudinal and time-series analyses, offering a dynamic perspective on the evolution of these anxieties over time (20).

In this study, we conducted an in-depth exploration of public attitudes towards anxiety disorders, specifically within the context of unemployment among Chinese university students, using the social media platform Weibo. By incorporating a temporal dimension into our analysis, we were able to track and understand how these attitudes have evolved over time. This multifaceted analytical approach allowed us to develop a comprehensive understanding of the intersection between youth unemployment and anxiety disorders within a digital context, shedding light on the societal, emotional, and mental health implications. This research significantly enhances our knowledge of the psychosocial impacts of unemployment on young adults and provides valuable insights that can help guide interventions and inform policies aimed at addressing these pressing issues. The importance of this study stems from its ability to capture real-time data on the nuanced and often transient mental states of young individuals facing economic hardships. By analyzing these expressions within a social media context, the research provides a unique perspective on the lived experiences and evolving sentiments of this vulnerable group. The findings illuminate both the immediate emotional responses to unemployment and the long-term trends and patterns that may influence societal and policy responses. Therefore, this study makes a significant contribution to the discourse on mental health and economic stability, emphasizing the urgent need for targeted mental health support and economic interventions specifically designed for the challenges faced by young graduates in today’s volatile job market.

## 2 Methods

### 2.1 Data collection

Sina Weibo, one of the foremost social media platforms in China, served as our primary data source for this investigation. Utilizing Sina Weibo’s application programming interface, we harvested posts containing the keywords “失业(unemployment) “, “焦虑(anxiety) “, and one of the 100 keywords related to university students (shown in Supplementary material) from January 1, 2019, to June 30, 2023. A total of 30,540 posts were collected and these originated from 22,415 unique accounts, indicating a discussion participation rate of approximately 0.005% among Sina Weibo users.

In order to optimally distill the textual characteristics and topical themes present in the posts, we instituted a comprehensive pre-processing protocol:

1) We eliminated posts generated automatically by organizational or Sina Weibo platform accounts.
2) References to user handles, signified by the “@” symbol, were removed.
3) All English letters and emoticons were expunged.
4) Using regular expressions, we filtered out the titles of topics and super topics, identifiable by the hashtag symbol “#” preceding and following the text, such as “#unemployment#.”
5) We identified and discarded posts that contained repetitive advertising content.
6) Posts that only contained keywords with little to no additional information were eliminated.

Through these meticulous pre-processing steps, we were left with 30,540 keyword-containing posts for analysis. To examine temporal trends in the volume of posts, we divided the posts into monthly cohorts.

Our research sourced its unemployment rate data from official statistics released by the National Bureau of Statistics of China. Considering that the age of university graduates is typically around 22 years old, we chose to focus on the monthly unemployment rates of individuals aged 16–24. This age bracket, we believe, provides the most pertinent snapshot of the employment situation confronting new graduates, allowing for a more accurate exploration of the relationship between unemployment rates and anxiety disorders among university students.

### 2.2 Methods of analysis

Our preprocessing phase led to the monthly categorization of posts, yielding a series dataset organized according to unemployment rates. For this analysis, we employed a linear regression model (executed in the R programming language; R Core Team, and the R Foundation for Statistical Computing) (21). The utilization of unemployment rate series analysis offered a robust mathematical framework for our dataset, enabling us to discern patterns and underlying information encapsulated within the temporal dimension of our sample.

Subsequently, we conducted a textual analysis to examine the linguistic features permeating the collected posts. Utilizing the Chinese psychoanalytic tool TextMind (22), we carried out a statistical analysis of high-frequency words that correlated with psychological attributes in the posts. TextMind, leveraging the Chinese lexicon C-LIWC (23), facilitates automatic word segmentation, word classification, and computation of frequency for each word category. C-LIWC comprises a lexicon that encapsulates 32 linguistic features, 32 psychological features distributed across six primary categories, and 38 additional attributes such as punctuation marks. In our study, we selected five major categories to scrutinize the trends and relevance characteristics inherent in each category. Furthermore, we chose 11 subcategories from these five major categories, along with four additional categories, to delve into the subtle changes present (shown in Table 1).

**Table 1:** The selected categories reflect mental characteristics, as derived from the linguistic inquiry and word count (LIWC) dictionary.

Category	Abbreviation	Examples
Social process	Social	Invite, hear, instruct, community, interact, public, culture
Family	Family	Son, daughter, husband, parents, uncle, cousin, family
Affective process	Affect	Serious, excessive, willing, rich, hope, promise, cope
Positive emotion	PosEmo	Affectionate, loving, welcome, praise, glorious, interesting, kind
Negative emotion	NegEmo	Resentful, heartless, failure, worry, trash, protest, abuse
Anxiety	Anx	Restless, impatient, insomnia, fright, panic, anxiety, nervous
Anger	Anger	Resentful, angry, enemy, fight, criticize, rage, agitated
Sadness	Sad	Pitiful, disappointed, inferior, sorrowful, suffering, helpless, sad
Biological process	Bio	Sick, fever, healing, tired, pain, numbness, vessels
Body	Body	Finger, skin, ear, perception, breath, eye, shirt
Health	Health	Live, care, insomnia, wound, surgery, health, scar
Cognitive process	CogMech	According, evidence, generally, intend, notice, otherwise, however
Insight	Insight	Exactly, seem, think, admit, notice, believe, feeling
Exclusive	Excl	Regardless, rather, if, otherwise, unless, suppose, however
Perceptual process	Percept	Say, show, watch, listen, feel, touch
Feel	Feel	Feel, soft, comfort, fuzzy, sharp, smooth, touch
(Others) I	I	I
(Others) assent	Assent	Hah-hah, alright, okay, yes, indeed, sure, clear, good
(Others) work	Work	Work, research, postgraduate, study, colleague, interview, unit
(Others) achievement	Achieve	Success, accomplish, achieve, encourage, reward, plan, effect

## 3 Results

### 3.1 Trend analysis of temporal fluctuations in urban youth unemployment rate

**Figure 1:**
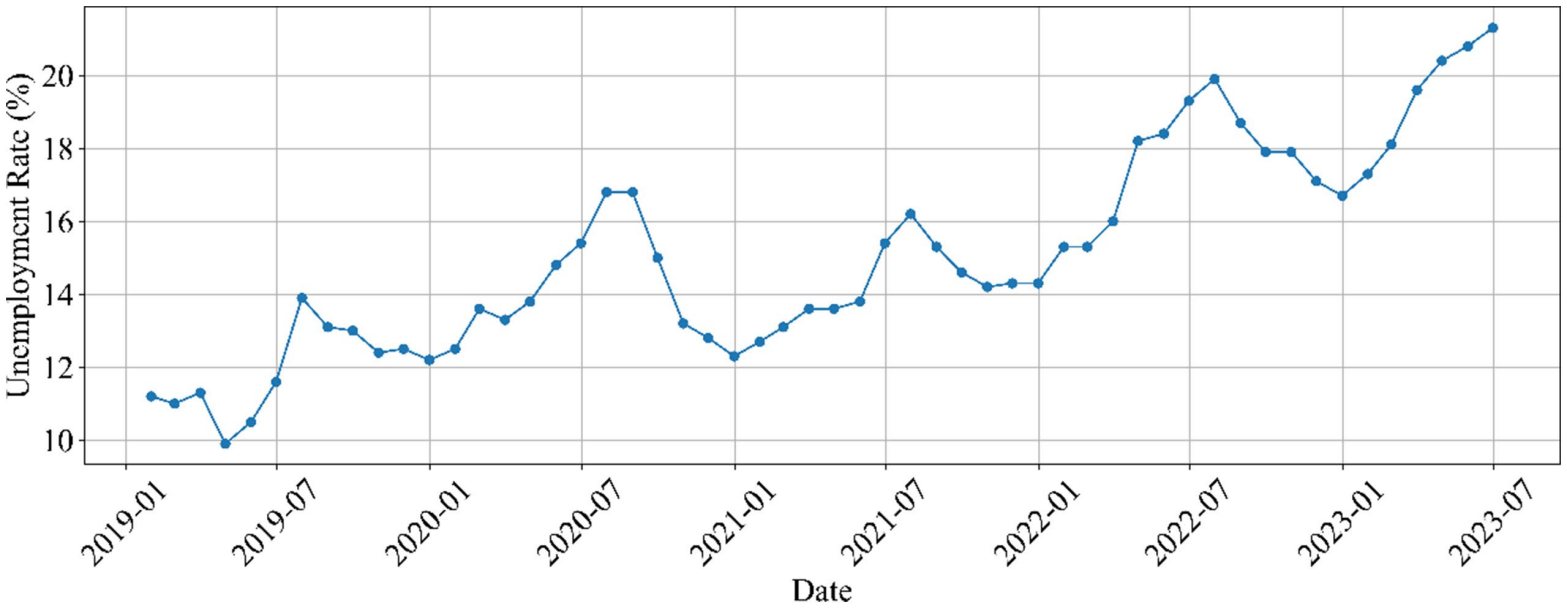
Urban unemployment rate for ages 16–24 in China (Jan 2019 - Jun 2023).

Between January 2019 and June 2023, the urban survey unemployment rates for China’s 16–24 age demographic have manifested a complex pattern, characterized by fluctuating dynamics (shown in Figure 1). The data series, expressed in percentages, commenced at 11.2% in January 2019, reaching a peak of 21.3% in June 2023. During this period, the unemployment rates experienced several noteworthy shifts, with discernible increases and subsequent declines, underscoring the volatile employment landscape for this age group. The emergence of the COVID-19 pandemic further exacerbated the unemployment situation during this period. The intricate interplay between the pandemic’s ramifications and the pre-existing employment dynamics underscores the multifaceted nature of the unemployment issue during this period. These data points, ranging from 9.9 to 21.3%, provide an intricate portrayal of the contemporary employment challenges facing young urbanites in China.

### 3.2 Trend analysis of the number of Sina Weibo posts

**Figure 2:**
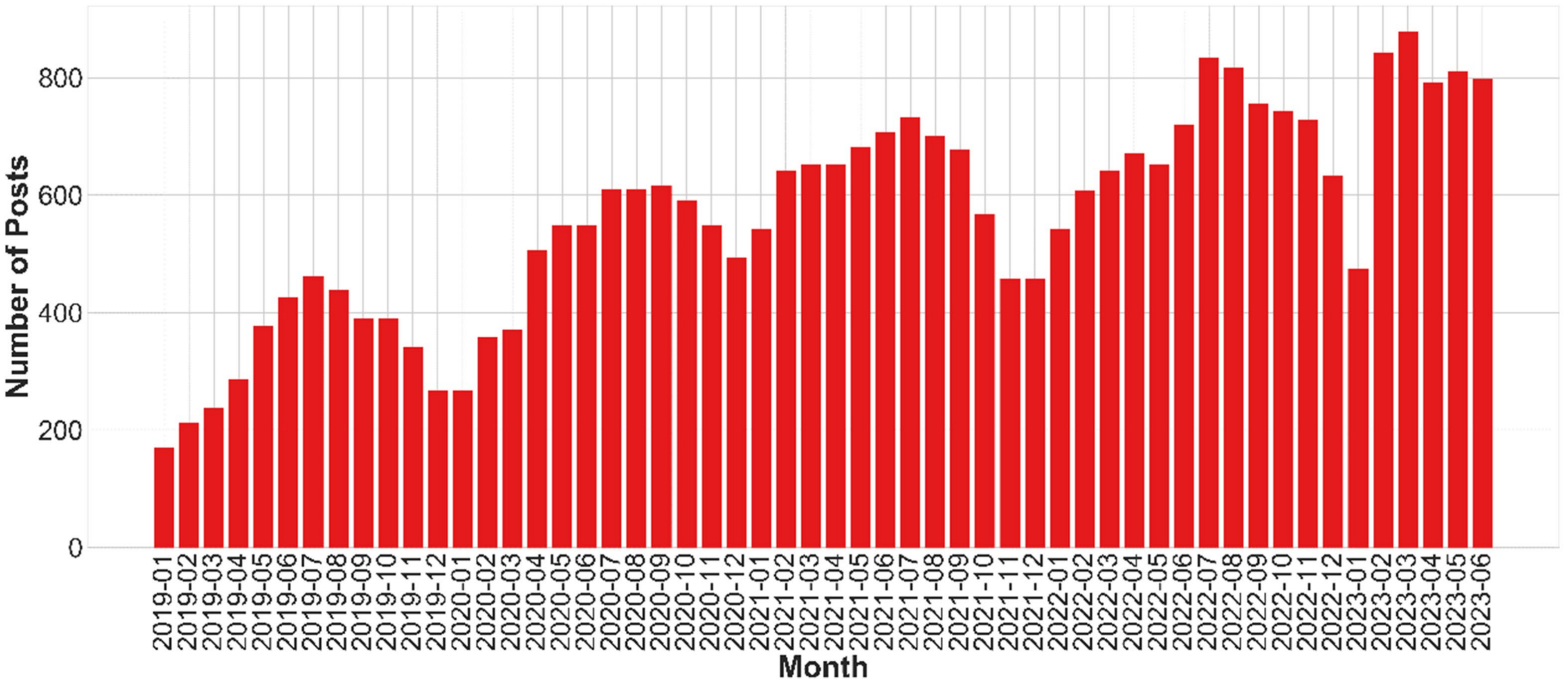
Number of posts related to college students’ unemployment anxiety.

In an attempt to gauge the sentiments of recent graduates towards unemployment, we harvested posts from Weibo, a widely used social media platform in China, spanning from January 2019 to June 2023. The data revealed an intricate trajectory of posts centered around unemployment-induced anxiety among university students. Commencing with 799 posts in January 2019, there was an observable escalation in such posts, peaking at 835 in April 2023. Notably, the volume exhibited several oscillations, indicative of the dynamic sentiment landscape. While the lowest post count recorded was 171, the highest reached an alarming count of 879. This digital barometer, chronicling the rising tide of anxiety over a span of four and a half years, underscores the pressing need to address employment concerns (Figure 2).

### 3.3 Analysis of the features of predominant categories and the features of subcategories of words

This study employs a robust linear regression analysis to discern the intricate relationships between predominant linguistic features and their corresponding subcategories. The Unemployment Rate is utilized as a pivotal socio-economic indicator to probing the interplay between language dynamics and macroeconomic factors. Five dominant linguistic features, namely Social, Affect, Bio, CogMech, and Percept, are meticulously scrutinized in conjunction with their associated subcategories. Strikingly, these linguistic facets unveil distinct correlations with the Unemployment Rate. Notably, Affect exhibits a noteworthy positive association (Estimate: 1.5e-04), indicating intensified linguistic manifestation as Unemployment Rates rise. Similarly, Bio establishes a significant yet measured connection (Estimate: 2.5e-05). Additionally, Social, CogMech, and Percept portray nuanced interdependencies, underscoring their sensitivity to socio-economic fluctuations (shown in Table 2).

**Table 2:** Linear regression analysis of the occurrence frequencies of the 5 predominant linguistic features and their respective 15 subcategories.

	Predictor	Estimate	SE	*t*-test	*R* ^2^	*p*-value
Predominant linguistic features						
Social^1^	Unemployment rate	−9.0e-05	2.37e-08	−3.8	0.28	0.001
Affect^2^	Unemployment rate	1.5e-04	2.941e-08	5.1	0.35	<0.001
Bio^3^	Unemployment rate	2.5e-05	1.0e-08	2.5	0.12	0.02
CogMech^4^	unemployment rate	2.8e-05	1.217e-08	2.3	0.15	0.03
Percept^5^	Unemployment rate	3.0e-05	1.111e-08	2.7	0.18	0.01
Subcategories						
Family	Unemployment rate	−2.0e-05	1.111e-08	−1.8	0.08	0.08
Positive emotion	Unemployment rate	−7.5e-06	5.357e-09	−1.4	0.05	0.17
Negative emotion	Unemployment rate	1.7e-04	2.742e-08	6.2	0.52	<0.001
Anxiety	Unemployment rate	1.1e-05	1.375e-08	0.8	0.03	0.42
Anger	Unemployment rate	8.0e-06	1.143e-08	0.7	0.02	0.48
Sadness	Unemployment rate	6.5e-05	1.512e-08	4.3	0.38	0.001
Body	Unemployment rate	−2.3e-05	6.970e-09	−3.3	0.25	0.002
Health	Unemployment rate	−1.2e-04	2.553e-08	−4.7	0.44	<0.001
Insight	Unemployment rate	−1.1e-05	5.238e-09	−2.1	0.10	0.04
Exclusive	Unemployment rate	2.2e-05	1.048e-08	2.1	0.16	0.04
Feel	Unemployment rate	7.5e-06	1.25e-08	0.6	0.01	0.55
I	Unemployment rate	1.6e-04	2.712e-08	5.9	0.53	<0.001
Assent	Unemployment rate	1.9e-05	8.636e-09	2.2	0.13	0.03
Work	Unemployment rate	−1.1e-05	5.238e-09	−2.1	0.10	0.04
Achievement	Unemployment rate	−1.4e-04	2.545e-08	−5.5	0.48	<0.001

^1^Social: social process. ^2^Affect: affective process. ^3^Bio: biological process. ^4^CogMech: cognitive process. ^5^Percept: perceptual process.

**Figure 3:**
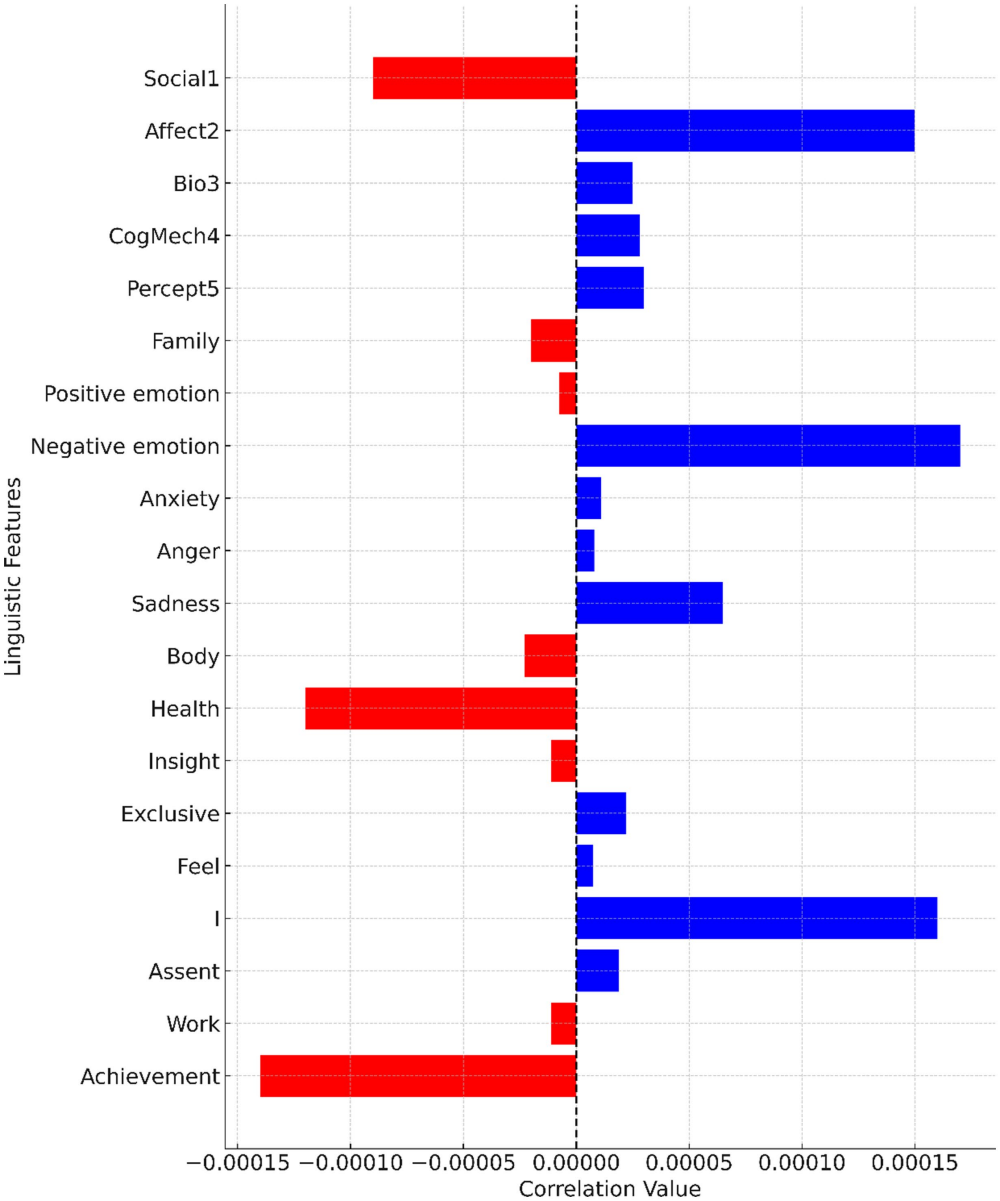
Correlation between linguistic features and unemployment rate.

Figure 3 visually represents the correlation between various linguistic features and the unemployment rate. The linguistic features are listed on the y-axis, while the x-axis showcases the correlation values. Bars extending to the right (in blue) indicate a positive correlation, suggesting that as the linguistic feature increases, the unemployment rate also tends to increase. Notably, features such as “Affect2” and “Negative emotion” show significant positive correlations. Conversely, bars extending to the left (in red) demonstrate a negative correlation, implying that an increase in the linguistic feature is associated with a decrease in the unemployment rate. Features like “Social1” and “Achievement” are prominent examples of this negative correlation.

Upon delving into the subcategories, compelling insights surface. Negative emotion registers a robust estimate (Estimate: 1.7e-04), illuminating its direct proportionality to unemployment rate—a testament to heightened emotional articulation during economic instability. Intriguingly, “I” and “Achievement” subcategories mirror positive correlations (Estimates: 1.6e-04 and −1.4e-04, respectively), hinting at linguistic markers of self-focus and personal attainment during unemployment episodes. Conversely, “Health” and “Body” subcategories exhibit negative correlations (Estimates: −1.2e-04 and −2.3e-05, respectively), suggesting linguistic reflections of physical and mental well-being considerations amid economic vicissitudes. Collectively, these findings paint a multifaceted portrait of linguistic responses to unemployment, offering nuanced insights into the emotional and psychological dimensions of individuals navigating economic flux.

## 4 Discussion

The present study employed a comprehensive approach to investigate the relationship between unemployment rates and anxiety expressions among university students in China. By analyzing data from Sina Weibo posts spanning from January 2019 to June 2023, the study uncovered intricate patterns and correlations between linguistic features and the fluctuating unemployment landscape. The investigation began by exploring the temporal trends in urban youth unemployment rates. The data indicated a complex and dynamic pattern, characterized by fluctuations that ranged from an initial 11.2% in January 2019 to a peak of 21.3% in June 2023. These variations underscore the volatile employment conditions faced by the 16–24 age demographic during this period. Notably, the months of June and July, often coinciding with the peak of the graduation season, played a significant role in the fluctuating dynamics. This period witnessed heightened expressions of anxiety and pressure, stemming from the impending graduation, as university students faced the challenges of entering the job market.

These fluctuations underscore the volatile employment landscape that defines this particular age group. The emergence of the COVID-19 pandemic significantly compounded the challenges faced by this demographic, particularly in terms of unemployment. The pandemic’s disruptive influence on various sectors of the economy acted as an accelerator for the upward trajectory of unemployment rates. Stringent lockdown measures, disruptions in supply chains, and diminished consumer spending collectively contributed to a contraction in economic activities, consequently leading to layoffs and a dearth of new job opportunities. This intricate interplay between the pandemic’s repercussions and the pre-existing dynamics of employment further emphasizes the multifaceted nature of the unemployment issue during this period. Within the scope of these intricacies, the data points spanning from 9.9 to 21.3% offer a compelling portrayal of the contemporary employment challenges confronted by young urban residents in China. This span of data not only reflects the magnitude of the challenge but also encapsulates the resilience and adaptability demonstrated by this age group amidst an evolving economic landscape. This finding aligns with broader discussions about the challenges of securing stable employment for young graduates, especially in the context of economic uncertainties.

The study delved into the sentiments expressed by university students regarding unemployment-induced anxiety using Sina Weibo posts. Over the course of four and a half years, the volume of posts relating to unemployment anxiety exhibited a dynamic trajectory, reaching its zenith at 835 posts in April 2023. This oscillating pattern reflected the evolving sentiments surrounding unemployment, highlighting the need to address the pressing concerns faced by recent graduates. This observation resonates with the notion that social media platforms provide a platform for individuals to voice their emotions and concerns, serving as a valuable resource for understanding public sentiment.

The findings of this study indicate a significant impact of unemployment rates on university students’ anxiety levels, particularly concerning their work and social life. The positive correlation observed between the “Affect,” “Bio,” “CogMech” and “Percept” linguistic category and the unemployment rate suggests that as unemployment rates increase, students express heightened negative emotions. This trend resonates with the notion that economic instability can lead to emotional distress, as individuals grapple with uncertainties surrounding their future prospects. In light of this correlation, the study’s data regarding the temporal trends in unemployment rates and corresponding anxiety expressions among students gain substantial relevance. The fluctuating dynamics of urban youth unemployment rates, ranging from 11.2% in January 2019 to a peak of 21.3% in June 2023, align with the observed trajectory of anxiety-related posts on Sina Weibo. This synchronicity highlights the real-time emotional responses of students to changes in unemployment rates, underscoring the interconnectedness of economic conditions and mental well-being. Furthermore, the study’s textual analysis of posts may provide insights into the specific anxiety triggers related to unemployment. Qualitative examination of the posts could reveal common themes such as fear of joblessness, financial concerns, and uncertainties about career trajectories. This information could enhance our understanding of the sources of anxiety and inform targeted interventions to support students during periods of economic instability.

The research also sheds light on the perception of discrimination and stigma against anxiety disorders among university students. The significant positive correlation between the “Negative emotion,” “Anxiety,” “Anger,” and “Sadness” subcategory and the unemployment rate suggests that students express more negative emotions as unemployment rates rise. This point reflects the broader societal context in which individuals with mental health challenges often confront negative attitudes and stereotypes. The data revealing high levels of discrimination perception, particularly in terms of self-denial and negative emotions, underscores the complexities of managing mental health in a cultural context that may not fully embrace open discussions about psychological well-being. The linguistic analysis’s correlation between linguistic features and the unemployment rate provides a lens through which to interpret these perceptions. This emotional response may contribute to the perception of discrimination, as heightened anxiety could amplify sensitivities to social judgments.

To delve deeper, future research could explore the specific context in which students perceive discrimination and stigma. Investigating narratives of personal experiences and societal interactions related to anxiety disorders could offer a richer understanding of the challenges students face. Additionally, interventions aimed at reducing stigma and promoting mental health awareness could be designed based on these insights. Additionally, future research could explore the development of machine learning techniques (24), such as ensemble learning algorithms (25), to more effectively capture linguistic features and textual sentiment. Furthermore, analyzing the spread of emotion through social networks (26) among users of social networking sites could be another promising direction.

## 5 Limitation

This study predominantly utilized data sourced from Sina Weibo, a leading social media platform in China. However, it’s important to note that this platform may not comprehensively represent the varied experiences and viewpoints of all university students. Social media platforms generally attract users who are more actively engaged online, which could introduce a selection bias. Furthermore, relying solely on data from Sina Weibo might not fully encompass the sentiments and experiences of university students, as some may prefer other platforms or offline methods to express their views.

Additionally, while this research extensively employed quantitative and linguistic analysis techniques, it did not integrate qualitative insights directly from the university students affected by unemployment and anxiety. Incorporating qualitative methods such as interviews or focus groups could significantly deepen the understanding of the students’ specific experiences, emotions, and perceptions. This approach would lend additional depth and context to the research findings, facilitating a more comprehensive exploration of the phenomena being studied.

## 6 Conclusion

In summary, this research conducted a comprehensive investigation into the interplay between unemployment rates, anxiety expressions, and linguistic features among university students in China. By analyzing data from Sina Weibo posts and official unemployment statistics, this study revealed intricate patterns and correlations that highlight the emotional and psychological dimensions of unemployment challenges.

The temporal trends analysis underscored the dynamic nature of urban youth unemployment rates, emphasizing the volatility of the employment landscape for the 16–24 age demographic. Concurrently, the sentiment analysis of social media posts illuminated the evolving emotional responses of university students towards unemployment-induced anxiety. These findings collectively emphasize the need for proactive measures to address the pressing concerns of recent graduates and foster a supportive environment.

The exploration of linguistic features demonstrated the nuanced relationship between language dynamics and macroeconomic factors. Notably, the positive correlation between the Affect category and unemployment rates emphasized the heightened emotional expressions during periods of economic uncertainty. Subcategories provided further insights, revealing how individuals cope and express themselves amidst challenges. As such, this study contributes to a holistic understanding of the multifaceted impact of unemployment on the emotional well-being of university students. Moving forward, the insights garnered from this research can inform policies and interventions that address both the economic and psychological aspects of unemployment, ultimately promoting the holistic well-being of young graduates in the face of adversity.

## Data availability statement

The raw data supporting the conclusions of this article will be made available by the authors, without undue reservation.

## Ethics statement

The study protocol was approved by the Guangzhou Sport University (2023LcLL-38). The studies were conducted in accordance with the local legislation and institutional requirements. Written informed consent for participation was not required from the participants or the participants’ legal guardians/next of kin because publicly available data: The research relied exclusively on data collected from publicly available posts on Sina Weibo, a social media platform. Since these posts are in the public domain and do not involve private or confidential information, obtaining individual consent from each user is not necessary.

## Author contributions

MT: Conceptualization, Data curation, Formal analysis, Writing – original draft, Writing – review & editing. ZW: Methodology, Resources, Writing – original draft. JL: Data curation, Formal analysis, Funding acquisition, Writing – review & editing. YL: Conceptualization, Investigation, Methodology, Writing – original draft. WL: Data curation, Methodology, Project administration, Writing – original draft.
