# Supplementary material for: Analyzing the impact of unemployment on mental health among Chinese university graduates: a study of emotional and linguistic patterns on Weibo
Source: Front Public Health. 2024 May 9;12:1337859. doi: 10.3389/fpubh.2024.1337859 (PMC11111880; doi:10.3389/fpubh.2024.1337859)
Supplement: Supplementary file 1 [file Data_Sheet_1.docx]

Supplementary Material

# Supplementary Data

We used the LLA model to generate 100 keywords about college students. The keywords are as follows:

“学士学位”,”本科学位”,”大一”,”毕业证书”,”学位证书”,”毕业典礼”,”学位授予”,”毕业设计”,”毕业论文”,”毕业照”,”毕业聚会”,”学术成就”,”实习经历”,”学生会”,”校友会”,”就业”,”招聘会”,”毕业旅行”,”毕业感言”,”学生评价”,”辅导员”,”指导老师”,”学业成绩”,”学分”,”毕业条件”,”培训”,”高校”,”毕业季”,”毕业典礼演讲”,”职业规划”,”人际关系”,”校园文化”,”专业选择”,”择业方向”,”大五”,”留学”,”创业”,”拓展技能”,”课外活动”,”学士服”,”学科竞赛”,”学生组织”,”社交网络”,”学习资源”,”学生宿舍”,”学生福利”,”毕业晚会”,”学士帽”,” 跨专业”,”本科生”,” 本科会议”,”毕业后计划”,”校友关系”,”荣誉证书”,”学术研究”,”实验室”,”课程安排”,”挂科”,”奖学金”,”助学金”,”贷款”,”学费”,”生活费”,”食堂”,”图书馆”,”校园活动”,”招生”,”录取通知书”,”学科专业”,”学术会议”,”研究项目”,”团队合作”,”学术期刊”,”学术道德”,”科研成果”,”发表论文”,”实践操作”,”实验技能”,”本科学历”,”教学方法”,”教学评估”,”学校排名”,”交换生”,”外语能力”,”大学生”,”大二”,”面试技巧”,”简历制作”,”工作经验”,”拓展课程”,”学术交流”,”毕业答辩”,”大四”,”大三”,”心理辅导”,”学生健康”,”学生安全”,”学生工作”,”学生社交”,”学生志愿服务”。
